# How Closely Related Are Parent and Child Reports of Child Alexithymia?

**DOI:** 10.3389/fpsyg.2020.588001

**Published:** 2021-01-08

**Authors:** Andrew J. Lampi, Vikram K. Jaswal, Tanya M. Evans

**Affiliations:** ^1^Department of Psychology, University of Virginia, Charlottesville, VA, United States; ^2^Center for Advanced Study of Teaching and Learning, School of Education and Human Development, University of Virginia, Charlottesville, VA, United States

**Keywords:** alexithymia, parent–child dyads, parent report, child report, self vs. other report

## Abstract

Alexithymia is a subclinical trait involving difficulty describing and identifying emotions. It is common in a number of psychiatric conditions. Alexithymia in children is sometimes measured by parent report and sometimes by child self-report, but it is not yet known how closely related the two measures are. This is an important question both theoretically and practically, in terms of research design and clinical practice. We conducted a preliminary study to investigate this question in a sample of 6- to 11-year-old neurotypical children and their parents (*N* = 29 dyads). Parent and child reports were not correlated, and 93% of parents under-estimated their child’s level of alexithymia relative to the child’s self-report. Based on these results, we hypothesize that when asked to report on the child’s alexithymia, children and parents may not be reporting on the same phenomenon, and thus these two measures may not be interchangeable. These provocative findings, however, must be considered preliminary: our analyses were sufficiently powered to detect a strong relation between the two types of report had one existed, but our analyses were not sufficiently powered to distinguish between a small relation and no relation at all.

## Introduction

Emotions shape social interactions in fundamental ways, contributing to our ability to predict and explain the behavior of ourselves and others. Difficulties in accurately interpreting emotions are common in a number of clinical conditions, from depression and anxiety (Honkalampi et al., 2000) to eating disorders (Morie and Ridout, 2018) to autism spectrum disorder (Berthoz and Hill, 2005). One theory is that the emotional difficulties experienced by individuals across these varied conditions stem from the same source—a subclinical trait called alexithymia (literally “without words for emotions”; Sifneos, 1973).

Alexithymia in adults is measured *via* a self-report questionnaire, most often the 20-item Toronto Alexithymia Scale (TAS-20; Bagby et al., 1994); individuals respond to questions that assess how they experience emotion (e.g., using a Likert scale: “I am often confused about what emotions I am feeling”). Even among adults without a diagnosed condition, about 10% of the general population experiences high levels of alexithymia (Honkalampi et al., 2000). Adults with high levels of alexithymia describe having difficulty with the identification of and/or discrimination between emotional states (Bagby et al., 1994). For example, people with high levels of alexithymia tend to have poor emotion recognition (Grynberg et al., 2012) and spontaneously imitate emotional displays less often than those with lower levels (Sonnby-Borgström, 2009). High levels of alexithymia also negatively correlate with emotional intelligence (Mikolajczak et al., 2007) and with measures of empathy (e.g., Luminet et al., 2011).

Not being able to recognize emotion in others or to match another’s emotional states or facial expressions may lead to less success in social interactions. In clinical descriptions, alexithymics are described as appearing uncomfortable or disinterested in social interactions (Berthoz et al., 2011). One theoretical proposal suggests that an inability to represent the emotional states of others–due to one’s own difficulty in identifying/discriminating between emotions–can lead to a breakdown in empathy and, thus, failed social interactions (Bird and Viding, 2014).

Researchers have recently begun investigating how alexithymia manifests in children and how it may impact their social interactions. In some studies, children’s level of alexithymia is measured using the Children’s Alexithymia Measure (CAM), a parent-report instrument that asks parents to report on their children’s putatively emotionally relevant behaviors (e.g., “How often does your child say ‘I don’t know’ when asked why he/she is upset”; Way et al., 2010). In other studies, children’s alexithymia is measured using the Toronto Alexithymia Scale for Children (TAS-C), with children reporting on their own internal experiences (e.g., “I find it difficult to say how I feel inside;” Rieffe et al., 2006). These two instruments are, to our knowledge, the only published instruments available for measuring children’s alexithymia in clinical and research settings.

Interestingly, studies that measure alexithymia in children using parent report *via* the CAM can produce apparently conflicting results with those using self-report *via* the TAS-C. For example, in Trevisan et al. (2016), participants between 7 and 13 years old were shown clips from animated children’s movies, and researchers coded participants’ spontaneous facial expressions. Higher levels of alexithymia in children as reported by parents on the CAM were associated with less emotional expressivity. Apparently different results come from a study using child self-report of alexithymia. In Wieckowski and White (2016), 9- to 12-year-old participants watched videos of actors producing particular emotional expressions and were asked to respond with a natural facial expression (i.e., how they would if they encountered the person in real life). Level of alexithymia children reported on the TAS-C was not related to how closely children matched the expression they saw in the videos. There were important differences between these two studies—for example, Trevisan et al. (2016) coded spontaneous facial expressions, whereas Wieckowski and White coded for the appropriateness of facial expressions in response to an actor’s expressions. But, the difference in findings raises the intriguing possibility that parent report and child self-report of alexithymia on the only two published instruments of which we are aware may not be interchangeable.

This is an important question because a common theoretical and practical concern when measuring a child-based construct is that parent and child reports of that construct may not be closely related. While children report on their own phenomenological experiences, parents must report on their *perception* of their child’s experiences (based on the child’s behavior). One might expect that this would mean self-report would always be preferred over parent report. But, children–especially young children–may lack the metacognitive ability to be able to report on their own experiences (e.g., Flavell, 1979; Schneider, 2008), in which case parent report would be required.

Sometimes the two reports are congruent. For example, parent and child reports of the child’s depression have been found to strongly correlate (*r* = 0.44; Eg et al., 2018). Additionally, the two types of reports sometimes share a similar factor structure—for example, in the case of the parent and child versions of the Children’s Depression Inventory (Kovacs, 1985), a commonly used measure of the child’s depression (Cole et al., 2000). This suggests that for some instruments, parents and children report on similar constructs, and these measures can be used interchangeably or in tandem.

But, there are also constructs where the parent and child reports of the child’s experiences do not match. For example, Kalvin et al. (2019) and Lagattuta et al. (2012) did not find a significant correlation between parent and child reports of the child’s level of anxiety (*r* = 0.02 and *r*s < 0.10, respectively; Lagattuta et al. (2012) compare parent and child reports over multiple subscales). Likewise, parents of children diagnosed with attention deficit/hyperactivity disorder tended to under-estimate (relative to the child report) their child’s self-esteem and mental health and over-estimate their physical function (Klassen et al., 2006). One explanation for these discrepancies could be the instruments themselves: poor psychometrics may lead to discrepant reports. However, the instruments used in these studies have been shown to have strong internal reliability (Spence, 1998; Klassen et al., 2006; Kalvin et al., 2019).

As children’s level of alexithymia is sometimes measured by parent report using the CAM and sometimes by child self-report using the TAS-C, it is essential to understand how closely the two measures are related. If both are measuring the same (or even related) construct(s), one would expect them to be correlated—that is, the higher the parent’s report *via* the CAM, the higher the child’s self-report TAS-C *via* the TAS-C. There is no standard correlation that researchers have agreed indicates that two measures ostensibly tapping into the same construct are actually doing so. But, a strong correlation–such as that of a magnitude of 0.5 or greater (Cohen, 1988)–would provide strong evidence that they were related. To our knowledge, only one study has compared parent and self-report of the child’s alexithymia CAM, though it was not the primary focus of their work. In Griffin et al. (2016), 8- to 13-year-olds and their parents each completed standardized questionnaires reporting on the child’s level of alexithymia CAM. Results showed a correlation of only 0.21 between parent report and self-report.

The study here had two primary goals and one exploratory goal. First, we investigated whether parent report of children’s alexithymia using the CAM and children’s self-report of alexithymia using the TAS-C are strongly related to each other, or whether, as suggested by Griffin et al.’s (2016) work, they are only weakly related. Second, we sought to investigate whether parents consistently over- or under-estimate their child’s level of alexithymia relative to the child’s own report.

Finally, adult measures of alexithymia and autism symptomatology AQ are strongly related, with correlations ranging from 0.44 to 0.72 (e.g., Berthoz et al., 2013; Aaron et al., 2015; Gökçen et al., 2016), and there is overlap in clinical descriptions of autism and alexithymia (e.g., Berthoz et al., 2011; Grynberg et al., 2018). Indeed, a burgeoning literature has suggested that alexithymia may be a better explanation than autism for several of the social differences thought to be characteristic of autism (Bird and Cook, 2013; Bird et al., 2010, 2011; Shah et al., 2016; Lassalle et al., 2018). If a parent has higher levels of alexithymia themselves (as measured by self-report), they may not be well-attuned to their children’s emotions or well-calibrated reporters of their children’s experience of emotion (e.g., Bird and Viding, 2014). Thus, a third goal was to conduct exploratory analyses to investigate whether parents with elevated levels of alexithymia or autism symptomatology are as calibrated with their child’s self-report of alexithymia as parents with low levels of these factors.

## Materials and Methods

### Participants

Thirty parent–child dyads participated in the study: 12 dyads (40%) consisted of mother–son pairs, 17 (57%) were mother–daughter, and 1 (3%) was father–son. The average age of the children was 8 years, 7 months (range: 6.0–11.11). The average age of the parents was 40.2 years (range: 33–49; *SD* = 4.97). Dyads were recruited as part of a larger study on parent–child interactions in autism, the clinical sample for which has yet to be collected, and the results of which will be presented elsewhere. Our sample size was determined by power analyses for the primary questions of the larger study, but G-power sensitivity analyses (Erdfelder et al., 1996) showed that our sample was large enough to detect a strong correlation (*r* ≥ 0.47) between parent and child reports with a power of 0.8, should a strong correlation exist. Participants were typically developing, primarily white, from middle-class backgrounds, and were recruited from a database of families who had previously expressed interest in participating in research in child development. This study was carried out in accordance with the recommendations of and approved by the Institutional Review Board for Social and Behavioral Sciences at the University of Virginia, protocol #2174. In accordance with the Declaration of Helsinki, parents gave written consent for their and their child’s participation; all children gave verbal assent, and all children above age seven gave written assent.

### Procedure

#### Parent Data

Parents completed the following questionnaires below.

##### The Children’s Alexithymia Measure (CAM; Way et al., 2010)

The CAM is a 14-item questionnaire in which parents report on their perceptions of their children’s level of alexithymia (see Appendix A). Parents use a four-point Likert scale to rate the frequency with which their child engages in certain behaviors, such as “Says ‘forget it’ or ‘leave me alone’ when asked about his/her feelings.” Higher scores are indicative of greater levels of alexithymia, although there is no “cut-off” to indicate clinical relevance. The maximum score is 42. The CAM has been found to have good internal reliability (Cronbach’s *α* = 0.92; Way et al., 2010).

##### Toronto Alexithymia Scale (TAS-20; Bagby et al., 1994)

The TAS-20 is a 20-item questionnaire that assesses adults’ perceptions of their own experience of emotion. Respondents rate on a five-point Likert scale how strongly they agree with statements, such as “I am able to describe my feelings easily” and “I don’t know what’s going on inside me.” The maximum score is 100. Individuals who score above 61 are considered “alexithymic,” those below 51 “non-alexithymic,” and scores between 51 and 61 are considered “borderline-alexithymic.” Scores on the TAS-20 were treated as continuous. The TAS-20 has demonstrated good internal reliability overall (Cronbach’s *α* = 0.86) and across subscales (0.71 ≤ Cronbach’s *α* ≤ 0.80; Parker et al., 2003).

##### The Autism-Spectrum Quotient Test (AQ; Baron-Cohen et al., 2001)

The AQ is a 50-item questionnaire designed to quantify the level of autism symptomatology in adults with or without a formal diagnosis of autism spectrum disorder. Participants report *via* a four-point Likert scale about how strongly they agree with statements about their own behaviors (e.g., “I prefer to do things with others rather than on my own”). The maximum score is 50. Scores above 32 are considered clinically relevant. The AQ has been found to have good test–retest reliability (*r* = 0.7) and good internal consistency within sub-domains (Baron-Cohen et al., 2001).

As this was part of a larger study, parents completed additional measures that are not reported here, including a developmental history/demographic information form and a parent-report measure of their child’s autistic symptomology (Autism-Spectrum Quotient—Child Version [Child-AQ]; Auyeung et al., 2008). Parents were presented questionnaires in a packet and were explicitly asked to complete them in the following order: History Form, CAM, Child-AQ, TAS-20, and Adult-AQ. This order was used to ensure that parents perceptions of their child’s experiences were not influenced by their responding to similar questions about themselves.

#### Child Data

##### Child self-report of alexithymia

We presented children with an adapted version of the Alexithymia Questionnaire for Children (here referred to as the TAS-C; Rieffe et al., 2006). The TAS-C was adapted from the TAS-20 described above (Bagby et al., 1994) and was standardized on a sample of 9- to 15-year-olds in Holland. The TAS-C has been found to have good internal consistency for two scales: difficulty identifying feelings (*α* = 0.73) and difficulty describing feelings (*α* = 0.75), but not for externally oriented thinking (*α* = 0.29; Rieffe et al., 2006). Despite this, in order to replicate previous studies using the TAS-C, we used the measure in its entirety. It has previously been used with participants as young as 6 years (Savarese et al., 2018).

The TAS-C was designed to be completed by children independently. But, given that our youngest participants were 6 years old and may not have been able to read independently, we had a researcher read the items aloud. Additionally, we were concerned that some of the TAS-C items were syntactically complex and/or used terminology that could be unfamiliar to our American participants (e.g., the use of “television programmes” and “films” as opposed to “T.V. shows” and “movies”). Thus, we changed the wording of 17 of the 20 items. The original and modified instruments are shown in Appendix B.

Children were seated at a table next to a researcher, who began the session with four questions designed to familiarize children with the instrument’s Likert scale. The researcher showed children a scale with three 2.7 × 2.4-inch rectangles with different amounts of shading and explained that “not like me” corresponded to the rectangle that was not shaded, “sometimes like me” to the rectangle with 50% gray shading, and “a lot like me” to the black rectangle. Children received four training items designed to elicit answers across the scale (e.g., if a child’s favorite and least favorite foods were pizza and broccoli, training items included “I really like to eat pizza” and “I really like to eat broccoli,” eliciting “a lot like me” and “not like me” responses). The researcher then read aloud the modified items shown in Appendix B, and children pointed to their responses using the scale.

Children’s responses were coded twice, once by the researcher during the session and independently by a coder from videotape. The coders agreed on 100% of responses.

We also piloted a measure on which children reported on their parent’s level of alexithymia. Several participants either failed to complete this portion of the session or answered in patterned ways (e.g., alternating extremes of the Likert scale) for the report-on-parent portion, and so we do not report data from this pilot measure here.

## Results

In what follows, we first provide the descriptive statistics of our sample. We then report on the relation between parent and child reports of children’s alexithymia using a correlational approach. Finally, we describe the relationship between parent report of child alexithymia and parent factors. Data were analyzed by R (version 3.5.1; R Core Team, 2020) using the RStudio interface (version 1.1.456; RStudio Team, 2020).

### Descriptive Statistics

To investigate whether there were any outliers in our data, we first examined the distributions for the CAM, TAS-20, AQ, and TAS-C. There was only one outlier: one parent’s report of their child’s level of alexithymia yielded a score more than three standard deviations above the sample’s mean. While the interpretation of our results remained the same whether this dyad was included or excluded, we chose to exclude them in order to prevent this score from having undue influence on our analyses. Thus, our final sample size was 29 dyads.

Table 1 shows the descriptive statistics for parent and child age and participants’ scores on our measures. As expected given that this was a typically developing sample, no participants reached clinical thresholds for alexithymia or autism: on the parent self-report of alexithymia (TAS-20), all but one parent scored in the “non-alexithymic” range (<51); on the parent self-report of autism symptomatology (AQ), all parents scored below the “clinically relevant” cut-off of 32. Finally, there are no cut-off scores available for parent report of child’s alexithymia (CAM) or child’s self-report of alexithymia (TAS-C); however, parents and children tended to report scores that were less than half of the maximum possible score.

**TABLE 1:** Descriptive statistics.

Statistic	Mean	St. Dev.	Min	Max
Parent age	40.17	4.97	33	49
Child age	8.55	2.01	6	11
**Parent measures:**				
TAS-20	36.38	7.66	24	52
CAM	5.86	4.98	0	18
AQ	14.72	5.50	6	24
**Child measures:**				
TAS-C	16.17	5.75	4	25
Parent-child discrepancy	−0.26	0.2	−0.6	0.23

*TAS-20, 20-Item Toronto Alexithymia Scale (Bagby et al., 1994); CAM, Children’s Alexithymia Measure (Way et al., 2010); AQ, Autism-Spectrum Quotient (Baron-Cohen et al., 2001); TAS-C, Toronto Alexithymia Scale—Child version (Rieffe et al., 2006). Parent–child discrepancy represents the child’s adjusted (actual/total score) self-report of alexithymia subtracted from the parent’s adjusted report of the child.*

### Comparing Children’s Self-Report of Alexithymia to Previous Work

We first conducted an analysis to confirm that results from our child self-report measure of alexithymia were comparable to results from earlier studies that used the measure on which ours is based. Recall that Rieffe et al. (2006) created the TAS-C by adapting the adult self-report measure (TAS-20), rewording items, and collapsing the Likert scale child participants used from five choices to three. As noted earlier, we made slight changes to the wording of several of the items used by Rieffe et al. (2006) and administered the instrument orally rather than by paper and pencil.

To confirm that these modifications did not meaningfully alter the instrument, we tested whether the two versions yielded similar distributions of total scores. We used as our comparison case a study by Griffin et al. (2016), who used the original TAS-C with a sample of 8- to 12-year-old British children and who confirmed that it had been administered without modification (C. Griffin, personal communication, January 31, 2019). We applied a bootstrapping procedure to the child data reported in Griffin et al. (2016), creating 10,000 random distributions using the mean, standard deviation, and size of the neurotypical sample reported in their study (*M* = 16.50, *SD* = 5.38, *N* = 32). Each bootstrapped distribution was compared with the distribution obtained on our adapted measure (*M* = 16.17, *SD* = 5.75, *N* = 29) using a Kolmogorov–Smirnov test. The bootstrapped distribution differed significantly from the obtained distribution on just 53 of the 10,000 simulations, or 0.53% of the time. In other words, our adapted child self-report alexithymia measure yielded a distribution very similar to the one obtained in Griffin et al. (2016), suggesting that our modifications in wording and delivery did not meaningfully alter the nature of the assessment.

### Comparing Parent and Child Reports of Child Alexithymia

#### Correlational Approach

**FIGURE 1:**
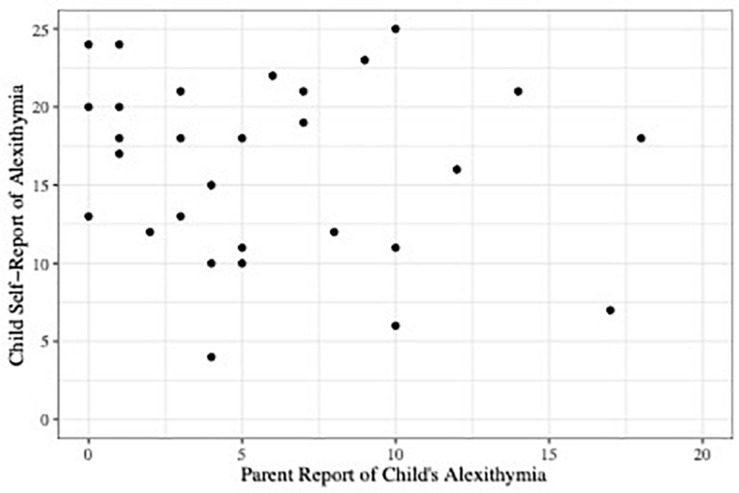
Scatterplot depicting parent report of child alexithymia on the CAM plotted against child self-report scores of alexithymia on the TAS-C.

Our primary goal was to determine how well parent report of the child’s alexithymia (CAM) matched the child’s self-report (TAS-C). Figure 1 shows a scatterplot of the relationship. As the figure shows and consistent with the findings reported by Griffin et al. (2016), the correlation between the two measures was low, *r*(28) = −0.14, 95% confidence interval (CI) [−0.48, 0.23], *p* = 0.46. We also conducted an equivalence test using the TOSTER package in R (Lakens, 2017) to investigate whether our observed correlation of *r* = −0.14 represented a weak correlation on the order of *r* = −0.20 (see Lakens et al., 2018). The test was neither significantly different from zero nor statistically equivalent to zero, *p* = 0.456. Thus, we are unable to draw conclusions regarding whether parent report and self-report of child’s alexithymia are only weakly correlated or not correlated at all.

This equivocation is the result of our analyses being underpowered to determine whether our detected correlation between the two types of report was different from zero. As noted earlier, a power analysis using G-power (Erdfelder et al., 1996) showed that with our sample size of 29 dyads, we would be able to detect a strong correlation (0.47 or higher) with a power of 0.80 had one existed in our sample. A *post-hoc* analysis using G-power showed that to conclude that our detected correlation of -0.14 was not different from 0 with 0.80 power would require a sample of approximately 400 dyads. Thus, while we are confident that there is not a strong relation between parent and child reports of the child’s alexithymia, we cannot say that they are not at all related.

#### Discrepancy Between Parent and Child Reports

Notwithstanding the power issues just described, we also investigated whether parents tended to under- or over-report their child’s alexithymia (relative to the child’s report). As Figure 1 shows, most parents reported low levels of alexithymia in their children: more than half of CAM scores were less than or equal to five (the CAM scale ranges from 0 to 42). Children’s TAS-C self-report scores covered a larger range, from 4 to 25 (the TAS-C scale ranges from 0 to 40). To analyze the discrepancy between CAM and TAS-C scores, we converted them to a common scale. For example, a child who scored 20 of 40 possible on the TAS-C received a score of 0.50; a parent who reported their child’s level of alexithymia was 10 of 42 possible on the CAM received a score of 0.24. The adjusted TAS-C score was subtracted from the adjusted CAM score to obtain a discrepancy score for that dyad. While neither of these could be considered to be an “objectively true” report, we have here chosen to use the child’s report as the reference point. Thus, a negative discrepancy score indicates that the parent under-estimated their child’s level of alexithymia relative to the child self-report, and a positive discrepancy score indicates that the parent over-estimated.

**FIGURE 2:**
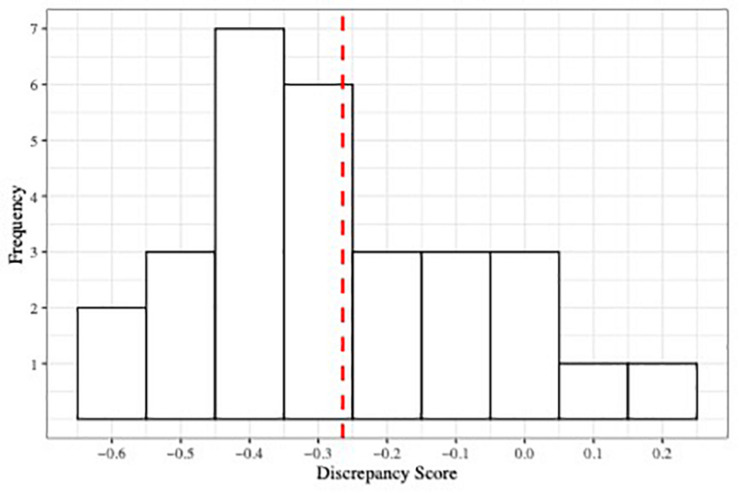
Histogram depicting the distribution of parent–child discrepancy score. Red dashed line indicates the mean of the distribution.

Figure 2 shows the distribution of discrepancy scores. As expected given Figure 1, parents tended to under-estimate their children’s alexithymia: the average discrepancy score was −0.26 (*SD* = 0.20), which, even when controlling for child age and gender, represents a significant difference from 0, *t*(26) = −2.27, 95% CI [−0.75, −0.05], *p* < 0.05, *d* = 0.421. Indeed, discrepancy scores were negative for 27 of 29 (93%) dyads.

### Relation Between Parent Report of Child Alexithymia and Parent Factors

We were also interested in whether the parent’s own level of alexithymia (as measured by the TAS-20) and their level of autism traits (as measured by the AQ) were related to their estimation of their child’s level of alexithymia (CAM). All three variables were positively correlated, though after Bonferroni correction (*p* = 0.05/3 = 0.017), none of them were significant: the CAM and TAS-20, *r*(28) = 0.37, 95% CI [0.0023, 0.65], *p* = 0.05; the CAM and AQ, *r*(28) = 0.31, 95% CI [−0.065, 0.61], *p* = 0.10; and the TAS-20 and AQ, *r*(28) = 0.41, 95% CI [0.046, 0.67], *p* = 0.03.

We conducted a linear regression predicting parent report of child’s level of alexithymia (CAM) score by the parent factors TAS-20, AQ, and the interaction between TAS-20 and AQ, covarying child age and child gender. The three variables of interest–scores on the CAM, TAS-20, and AQ–were scaled using z-transformations. The resulting model was not significantly different from the null model, *F*(5,23) = 1.65, *p* = 0.19, adjusted *R*^2^ = 0.10, and neither the TAS-20, AQ, nor their interaction were significant predictors (see Table 2 for coefficients and 95% CIs). Thus, parents’ self-reported levels of alexithymia and autism symptomatology did not predict their ratings of their typically developing children’s alexithymia.

**TABLE 2:** Regression results predicting CAM from parent-based factors.

	*Dependent variable*
	CAM
Child Gender (Male)	0.392 (−0.378, 1.161)
Child Age	−0.089 (−0.296, 0.118)
TAS-20	0.281 (−0.131, 0.693)
AQ	0.177 (−0.274, 0.628)
TAS-20 * AQ	0.200 (−0.409, 0.810)
Constant	0.525 (−1.365, 2.414)
Observations	29
*R*^2^	0.264
Adjusted *R*^2^	0.103
Residual Std. Error	0.947 (df = 23)
*F* Statistic	1.646 (df = 5; 23)

*Regression coefficients are presented in the format: β (95% confidence intervals of β).*

## Discussion

Our study had two primary goals. The first was to investigate the relation between a widely used parent report measure of their child’s level of alexithymia (CAM) and the child’s self-report of their own level of alexithymia (TAS-C). If the two measures are tapping into the same construct, one might expect them to be strongly correlated (i.e., a correlation of 0.5 or above). Replicating a secondary finding reported by Griffin et al. (2016), however, we detected only a small correlation. Our second goal was to investigate whether parents under- or over-estimated their child’s report of their alexithymia. Analyses revealed that most parents under-estimated their child’s level of alexithymia relative to the child’s own report. Together, these findings may help to explain why some studies find a relation between pediatric levels of alexithymia and performance on emotionally relevant tasks (Trevisan et al., 2016), whereas others do not (Wieckowski and White, 2016): it may depend, at least in part, on who the reporter is.

Crucially, however, our findings must be considered in light of two important limitations. First, the data reported here were collected as part of a larger study investigating parent–child relations in autism; the goal of that larger study was not to investigate questions about parent vs. child report of child’s alexithymia. Our sample size was similar to that used in the study by Griffin et al. (2016), which also reported a non-significant correlation between parent and child reports of the child’s alexithymia. However, as described above, our correlational analyses [and those of Griffin et al. (2016)] were underpowered to be confident that the correlation between parent and child reports was zero.

A power analysis suggested that it would require 400 dyads to be certain there was no correlation between parent and child reports of child’s alexithymia (i.e., that the correlation between the CAM and the TAS-C was not different from 0). There are two aspects of this power analysis that we should make explicit. First, it is of course based on the assumption that the distribution of scores from our sample of 29 dyads was representative of the population. If the distribution of scores from our sample was not representative, then our power analysis would have under- or over-estimated the number of dyads required. Second, ours was a neurotypical, low-alexithymia sample. It is possible that a nonlinear relationship exists between parent and child reports of alexithymia (i.e., evident in those who score in the clinical ranges on these instruments, but not among those who do not). For a sample including respondents who fall at more extreme ends of these distributions, the number of dyads necessary to reliably detect (or fail to detect) a correlation between parent and child reports may be different.

Another approach to analyzing the relationship between parent and child reports of child’s alexithymia would be to use Structural Equation Modeling (SEM), which has an important advantage over the correlational approach we used. Whereas our correlational approach operated across summed scores from our instruments, SEM can weigh individual items on each instrument differently based on how strongly they predict latent variables (Maruyama, 1998; Boker, 2018). As the CAM and the TAS-C include different items, this could be beneficial: examining the covariance between the latent variables from the two instruments could show how the constructs underlying each one relate, which is not possible with the correlational method. Our sample of 29 dyads is too small to conduct an SEM analysis. But, a Monte Carlo simulation we conducted with our data (*via* the OpenMx package from Neale et al. (2016) and based on recommendations provided in Muthén and Muthén (2002); Meuleman and Billiet (2009), and Wolf et al. (2013)) suggested that the sample size of 400 dyads recommended for the correlational approach and described above would also be sufficiently powered for an SEM analysis.

If future, appropriately powered analyses show that the parent and child perceptions of the child’s alexithymia are not related, there could be at least three explanations. The first may have to do with the instruments themselves: items on the parent-reported CAM focus primarily on the child’s behaviors (e.g., “Physically removes self from situations when asked to talk about feelings” and “Uses few words to describe most of his/her feelings”); only a few questions pertain to parent perceptions of the child’s internal experiences (e.g., “Has difficulty saying he/she feels sad/happy even though he/she looks sad/happy”). In contrast, most items on the child-reported TAS-C ask explicitly about the child’s introspective experience of emotion (e.g., “If I am upset, I don’t know if I am sad, scared, or angry”; “If I’m angry, I often don’t know why”; and “It’s hard for me to say how I really feel inside, even to my best friend”). The behaviors that parents report on when completing the CAM are presumably intended to serve as indications of the kinds of internal experiences the child him/herself reports on the TAS-C. However, the CAM items may not, in fact, be very good proxies for children’s responses to TAS-C items.

A second explanation could be that children did not understand the scale or items on the TAS-C, in which case, their responses would be uninterpretable (and their overall alexithymia score would not be expected to correlate with the score reported by their parents). We think that this is unlikely as we provided pre-training on how to use the scale, and because Rieffe et al. (2006) found that the original version of the TAS-C had the same factor structure as the TAS-20 and correlated with scores of somatic complaints (a measure of the instrument’s predictive validity). Likewise, we found that our sample’s distribution of TAS-C scores did not significantly differ from that collected by Griffin et al. (2016), who used the original, unmodified TAS-C. This suggests that our sample, despite having received a modified instrument and delivery method, did not answer in a way discrepant from previous samples and understood both the items and the scale used to answer them.

A final explanation is that parents were not accurately reporting on their children’s behaviors. We attempted to explore this possibility with our third, exploratory goal by investigating whether parents’ self-reported levels of alexithymia and autism symptomatology were related to their reports of their children’s alexithymia. We were interested in whether parents’ level of alexithymia was related to their ability to report on their child’s level of alexithymia because one of the difficulties associated with higher levels of alexithymia is in recognizing and interpreting how others feel (Moriguchi et al., 2006; Bird and Viding, 2014; Grynberg et al., 2018). Thus, parents higher in alexithymia might have more difficulty reporting on their child’s level of alexithymia. Because previous work has found a relation between alexithymia and autism symptomatology in adults (e.g., Berthoz et al., 2013; Aaron et al., 2015; Gökçen et al., 2016; but also see Hobson et al., 2018), we also considered the possibility that parents higher in autism symptomatology would have more difficulty reporting on their child’s level of alexithymia. We found no relation between parent levels of alexithymia and autism symptomatology and neither predicted parent report of child alexithymia, though these analyses were also underpowered.

In addition to parent alexithymia and autism symptomatology, there are a number of other parent-based factors that might also influence their reports of the child’s alexithymia. Future work may wish to account for, for example, parental symptoms of depression. As noted in the Introduction section, high levels of alexithymia are observed in a number of other diagnoses, including depression (Honkalampi et al., 2000). Higher levels of maternal depression have been associated with lower scores on parental sensitivity (NICHD Early Child Care Research Network, 1999), which could include sensitivity toward their child’s emotions. An important question would therefore be to determine whether any discrepancies in parent versus child reports are predicted by parental alexithymia and/or depression.

If parent and child reports of the CAM are unrelated (or are only weakly related, as our data suggest), there are a number of practical implications. First, it would suggest that the two ways of measuring alexithymia ought to be used in research and clinical settings in theoretically driven ways, rather than simply for ease or convenience of administration. Second, it implies that failing to match the assessment to the information needed could have negative therapeutic implications. For example, if one were interested in addressing the child’s apparent distress and frustration over difficulties expressing emotion, using the parent’s perception of their child’s emotional experiences, *via* the CAM, would be called for. But, if one were interested in helping the child understand their emotional experiences and needed to gauge therapeutic progress, measuring the child’s construct of their alexithymia *via* the TAS-C would be appropriate. Crossing these, however, could lead to clinicians having an inaccurate understanding of a client’s progress. Thus, practitioners will want to carefully select which assessment to use, ensuring that the intervention is associated with the proper source of information.

### Limitations

There were a number of limitations to this study. First, as has been noted, ours was a small, homogenous, non-clinical sample, and the data had little variability. This contributed to our diminished statistical power and may limit the generalizability of our findings. Future studies should recruit a larger, more diverse sample, especially with parents and children with higher levels of alexithymia and autism symptomatology. This would increase the generalizability of our findings and would allow for more in-depth analysis of parent-based factors that might influence their reports of child’s alexithymia, such as autism symptomatology or parental alexithymia.

Second, researchers interested in investigating the relationship between parent and child reports of child’s alexithymia should consider developing instruments where the same questions are asked of both reporters (see also Griffin et al., 2016). Naturally, when doing so, only the children would be able to “accurately” self-report on their internal experiences. However, asking parents and children the same questions could help determine whether any discrepancies between reports arise because parents are being asked to report on a separate construct from what children are being asked to report on, or if parents’ perceptions of their children’s internal states truly do not align with their children’s actual experiences.

Third, an important question is whether the relation between parent and child reports of child’s alexithymia varies between mothers and fathers. In our study, only one dyad included a father, and thus we are unable to investigate this question. This will be an important question for future research.

Finally, our study collected only questionnaire data as a means of determining whether parents’ perceptions of their children’s experiences are different from children’s self-reports of those experiences. Future work on this topic should include observational measures that characterize the parent–child dyad, such as using free-play paradigms that can be coded for parental sensitivity. When using parent/child reporting discrepancies as the sole metric of interest, it becomes impossible to determine whether any apparent discrepancies are the result of instrumentation or more generalized parental insensitivity. Including an additional measure, such as a behavioral paradigm, could help tease apart this distinction, allowing one to have more confidence in whether the instrument or parental sensitivity led to observed discrepancies.

## Conclusion

We investigated the relationship between a parent-report measure and a child self-report measure of the child’s alexithymia. In our sample of neurotypical children and parents, we found that the two measures were not correlated, that parents significantly under-estimated their children’s alexithymia, and that parent estimates of child alexithymia were not predicted by any parent-based factor we measured.

Our analyses were sufficiently powered to detect a correlation between parent and child reports of the child’s alexithymia of at least 0.47 or greater–with 0.8 power–had one been present. However, we found a correlation of just −0.14. While we cannot conclude that these two reports are not related at all, our results suggest that these measures do not share a strong relationship. Arguably–as a clinician or researcher–before assessing parent and child reports of the child’s alexithymia, one would want to use two measures that correlate with a stronger effect.

## Data Availability Statement

The raw data supporting the conclusions of this article will be made available by the authors, without undue reservation. The data reported in this paper and R code on which analyses are based are available at https://osf.io/aqkf4/?view_only=04b2842877524e08b565d081a82fd5ba.

## Ethics Statement

The studies involving human participants were reviewed and approved by the Institutional Review Board for Social and Behavioral Sciences at the University of Virginia. Written informed consent to participate in this study was provided by the participants’ legal guardian/next of kin.

## Author Contributions

AL aided in the study design, the data collection, the data analysis, and manuscript drafting. VJ aided in the study design, the data analysis, and manuscript drafting. TE aided in the study design, the data analysis, and manuscript drafting. All authors contributed to the article and approved the submitted version.

## Conflict of Interest

The authors declare that the research was conducted in the absence of any commercial or financial relationships that could be construed as a potential conflict of interest.

## Appendix A

**TABLE A1:** Items from the children’s alexithymia measure (CAM; Way et al., 2010).

	**CAM**
Item 1.	When asked about how he/she is feeling, instead talks about what he/she has been doing.
Item 2.	Has difficulty saying he/she feels sad even though he/she looks sad.
Item 3.	Talks about unimportant things/topics instead of sharing his/her feelings.
Item 4.	Has long periods of little or no emotional expression, interrupted by bursts of emotional expression.
Item 5.	Has difficulty saying he/she is happy even though he/she looks happy.
Item 6.	Physically removes self from situations when asked to talk about feelings.
Item 7.	Makes up unrelated stories when asked about his/her feelings.
Item 8.	Verbal expressions of feelings do not match non-verbal expressions of feelings.
Item 9.	Changes the topic of conversation when asked about his/her feelings.
Item 10.	Has difficulty naming his/her positive feelings (such as joy, happiness, excitement).
Item 11.	Says “forget it” or “leave me alone” when asked about his/her feelings.
Item 12.	Has trouble finding words or getting words out when talking about his/her own feelings.
Item 13.	Uses few words (may just be “good”/”bad”) to describe most of his/her feelings.
Item 14.	Says “I don’t know” when asked why he/she is upset.

## Appendix B

**TABLE A2:** Comparing items on the children’s version of the Toronto Alexithymia Scale (TAS-C original; Rieffe et al., 2006) and the TAS-C modified.

	TAS-C Original	TAS-C Modified
Item 1.	I am often confused about the way I am feeling inside.	I am confused about the way I feel inside a lot of the time.
Item 2.	I find it difficult to say how I feel inside.	It is hard for me to say how I feel inside.
Item 3.	I feel things in my body that even doctor’s don’t understand.	I feel things in my body that other people don’t understand.
Item 4.	I can easily say how I feel inside.	It is easy for me to say how I feel inside.
Item 5.	When I have a problem, I want to know where it comes from and not just talk about it.	If I have a problem, I want to know where it comes from and not just talk about it.
Item 6.	When I am upset, I don’t know if I am sad, scared, or angry.	If I am upset, I don’t know if I am sad, scared, or angry.
Item 7.	I am often puzzled by things that I feel in my body.	I am often unsure about things that I feel in my body.
Item 8.	I’d rather wait and see what happens, instead of thinking about why things happen.	I like seeing things happen instead of thinking about why they happen.
Item 9.	Sometimes I can’t find the words to say how I feel inside.	Sometimes I can’t find the words to say how I feel inside.
Item 10.	It is important to understand how you feel inside.	It is important to understand how you feel inside.
Item 11.	I find it hard to say how I feel about other people.	It is hard for me to say how I feel about other people.
Item 12.	Other people tell me that I should talk more about how I feel inside.	Some people tell me I should talk more about how I feel inside.
Item 13.	I don’t know what’s going on inside me.	I don’t know what’s going on inside me.
Item 14.	I often don’t know why I am angry.	If I’m angry I often don’t know why.
Item 15.	I prefer talking to people about everyday things, rather than about how they feel.	I like to talk to people about things they do instead of how they feel.
Item 16.	I prefer watching funny television programmes, rather than films that tell a story about other people’s problems.	I like watching funny t.v. shows, more than t.v. shows about people’s problems.
Item 17.	It is difficult for me to say how I really feel inside, even to my best friend.	It is hard for me to say how I really feel inside, even to my best friend.
Item 18.	I can feel close to someone, even when we are sitting still and not saying anything.	I don’t have to talk or do something with another person to feel close to them.
Item 19.	Thinking about how I feel, helps me when I want to do something about my problems.	When I want to do something about my problems, thinking about how I feel helps.
Item 20.	When I have to concentrate on a film to understand the story, I enjoy the film much less.	I don’t like movies where I have to concentrate to understand the story.
